# Critical Aspects in the Preparation of Extemporaneous Flecainide Acetate Oral Solution for Paediatrics

**DOI:** 10.3390/pharmaceutics13111963

**Published:** 2021-11-19

**Authors:** Antonella Casiraghi, Giorgio Centin, Francesca Selmin, Claudia Picozzi, Paola Minghetti, Davide Zanon

**Affiliations:** 1 Department of Pharmaceutical Sciences, Università degli Studi di Milano, 20133 Milan, Italy; francesca.selmin@unimi.it (F.S.); paola.minghetti@unimi.it (P.M.); 2 Pharmacy and Clinical Pharmacology Department, Institute for Maternal and Child Health IRCCS Burlo Garofolo, 34137 Trieste, Italy; giorgio.centin@studenti.unimi.it (G.C.); davide.zanon@burlo.trieste.it (D.Z.); 3 Department of Food, Environmental and Nutritional Sciences, Università degli Studi di Milano, 20133 Milan, Italy; claudia.picozzi@unimi.it

**Keywords:** off-label, age-related dose, extemporaneous preparation, oral delivery, solubility, compatibility, stability, preservative

## Abstract

The availability of liquid oral preparations compounded by pharmacists is essential to meet paediatric needs which remain unanswered by the pharmaceutical industry. Unfortunately, compendial monographs are often not available and, in many cases, pre-formulation studies (e.g., compatibility with other excipients and solubility evaluations) are not performed in-depth, leading, in some rare cases, to the inadvertent administration of a toxic dose. In this study, the preparation of an oral liquid formulation for paediatric use, containing flecainide acetate at different strengths, was considered, taking into account the possible effects of conventionally used excipients. First, the optimal vehicle was selected based on a solubility study, evidencing some unexpected formations of precipitates. As a matter of fact, the buffers commonly used for oral solutions significantly reduced flecainide solubility, and the concomitant presence of citrate buffer and methylparaben even caused the formation of non-resuspendable crystals. Then, chemical, physical, and microbiological stability were assessed. Solutions at strengths of 10 and 20 mg/mL flecainide acetate were stable up to 8 weeks when compounded by using a 40% sucrose solution as a vehicle. Microbiological data showed that the use of methylparaben was not necessary over this time period.

## 1. Introduction

In order to address the unmet needs of paediatric patients, which are very often not satisfied by authorised medicinal products available on the market [1,2,3], magistral formulae can be extemporaneously prepared in pharmacy settings. In this case, when compendial monographs are not available or applicable, safety and efficacy become strictly related to the correct use of each component, the calculations performed, the accuracy and precision of weights and volumes, compliance with the compounding procedures, and appropriate operating conditions [4,5]. Moreover, as further control, the compounding activity requires written standard operating procedures (SOPs), well-controlled equipment, and harmonised regulations [6]. Ultimately, the source of the active substance may require a specific evaluation, being not always available as the pure active ingredient used in industrial production, but rather necessitating the manipulation of a finished dosage form, such as tablets, capsules, or solutions [3,7]. Therefore, in order to ensure the required quality attributes and prevent the risk of compromising the treatment, the compounding activity in a pharmacy setting should be based on an experimental study aimed to verify the suitability [8,9] other than the stability of the preparation. 

Flecainide acetate (FlAc), a class IC antiarrhythmic agent (according to the Vaughan-Williams classification) with good absorption after oral administration and good solubility (BCS Class 1) [10], is an interesting case. The drug is commonly used in paediatric patients to treat tachyarrhythmias, and it is also very effective for the treatment of foetal tachyarrhythmias [11] as well as for the maintenance of sinus rhythm in children with or without structural heart disease [12]. Despite the British National Formulary for Children supporting the administration of FlAc to paediatric patients from 1 month to 17 years of age [13], none of the authorised dosage forms (50–150 mg oral tablets and 10 mg/mL intravenous injectable solution) are intended for use in children under 12 years. Moreover, due to children’s growth, dosing must be continuously adjusted according to clinical response and the plasma concentration of flecainide (Fl) [13] and, to this purpose, liquid formulations are extemporaneously prepared [14]. The literature reports very few examples of oral solutions [13,15,16,17,18], mainly at a concentration of 20 mg/mL, obtained by mixing pure FlAc [15] or finely crushed tablets [16,17,18] with a limited number of excipients or, alternatively, with commercially available unmedicated oral liquid vehicles. Generally speaking, the formulation of an oral liquid should take various aspects into consideration, i.e., pH, osmolarity, viscosity, the presence of preservatives, and palatability. Each of these attributes is related to the use of specific excipients and, therefore, pose their own incompatibility or instability issues. As an example, consideration should be given to any data regarding the effect of pH on the solubility and stability of the drug. In the case of extemporaneously prepared oral liquids of FlAc, only one is reported to be transparent [15], even though the drug concentration is always far below its solubility limit, suggesting a possible physical incompatibility with the vehicle. Although it is possible to administer the correct dose even from suspensions [17,18], it should be noted that the presence of a precipitate that has not been properly characterised could affect stability and bioavailability in unexpected ways. Indeed, the literature reports at least one case of intoxication due to the formation of Fl crystals [19].

In this paper, oral liquid formulations for paediatric use, containing different strengths of FlAc, were investigated, aiming to assess the influence of conventionally used excipients—namely cosolvents, preservatives, and buffers. The availability of concentrations lower than 20 mg/mL may be very useful for clinicians [13], especially when FlAc is prescribed to very young children (including new-borns and toddlers) and low-weight patients.

## 2. Materials and Methods

### 2.1. Materials

FlAc pure powder was supplied by Farmalabor Srl (Canosa di Puglia, Italy). 

Components of the oral formulations: Milli-Q^®^ ultrapure water was used; sucrose and monosodium phosphate were supplied from VWR International Srl (Milan, Italy); citric acid and glycerol were bought from ACEF Spa (Fiorenzuola d’Arda, Italy); all other materials were supplied by Carlo Erba Reagents Srl (Cornaredo, Italy).

The commercially available suspending vehicle OraPlus^®^ (composition: microcrystalline cellulose, sodium carboxymethylcellulose, xanthan gum, carrageenan, potassium sorbate, methylparaben, sodium phosphate, citric acid, simethicone, purified water) [20] and the flavoured syrup vehicle OraSweet^®^ (composition: sucrose, glycerine, sorbitol, citrus berry flavour, methylparaben, potassium sorbate, citric acid, sodium phosphate, purified water) [20], produced by Paddock Laboratories LLC (Minneapolis, MN, USA), were imported by the IRCCS Burlo Garofolo hospital pharmacy.

All solvents were of analytical grade unless specified.

### 2.2. Preparation of Oral Solutions

The composition of each vehicle is reported in Table 1. Each component was accurately weighed, transferred into a beaker, then dissolved in water by magnetic stirring. Methylparaben was solubilised in a portion of total water before addition. 

For stability evaluation, 10 and 20 mg/mL solutions were prepared, adding FlAc and stirring until complete dissolution. 

All preparations were stored in tight glass vials protected from light.

### 2.3. Determination of Solubility

The solubility of FlAc in the aqueous vehicles reported in Table 1 and in commercially available oral suspending vehicles was determined by the shake flask method. Briefly, an excess quantity of the drug substance was added to 1 mL of each test solution and left under magnetic stirring at 25 °C for 24 h. After that, the solution was filtered (0.45 µm H-PTFE membrane, Merck KGaA, Darmstadt, Germany), diluted in the mobile phase, and analysed by HPLC for the determination of FlAc content. Solubility was calculated as the mean of three replicates.

The sediment, when possible, was isolated and characterised according to “Precipitate isolation and characterisation”, Section 2.5.

### 2.4. Chemical and Physical Stability Study

Preliminary stability tests on 10 mg/mL strength were carried out using F2–F6 as vehicles. If precipitation occurred, the sediment was isolated and characterised according to “Precipitate isolation and characterisation”, Section 2.5; moreover, the supernatant was tested for drug content after filtration.

Using the most appropriate vehicle (F3), the chemical stability of 10 and 20 mg/mL FlAc solutions were was monitored over an eight-week period on samples stored at 4 ± 1 °C (Labor 2T 500 ECT-F Touch, Fiocchetti Snc, Luzzara, Italy), 25 ± 1 °C (standard heating oven 160, Memmert GmbH, Schwabach, Germany), and 40 ± 1 °C (INCU-Line IL 53, VWR International Srl, Milan, Italy). For each temperature condition, samples were prepared in triplicate.

After vigorous shaking, aliquots were withdrawn at predetermined intervals (0, 14, 28, 42, 56 days), and tested for pH (InLab Expert Pro-ISM, Mettler-Toledo Spa, Milan, Italy) and drug content. The formulations were also checked for visual appearance. Solutions were considered stable if no precipitation occurred and the mean drug concentration was found within the range of 90–110% of the labelled concentration, with a 95% confidence interval.

### 2.5. Precipitate Isolation and Characterisation

The formed precipitate was separated using filter paper, washed, and dried until a constant weight was reached. The precipitate was characterised by Fourier-transform infrared (FT-IR) spectroscopy, differential scanning calorimetry (DSC), and HPLC.

### 2.6. Differential Scanning Calorimetry and Infrared Studies

DSC was performed by using a DSC1 Star System (Mettler-Toledo Spa, Milan, Italy). After being accurately weighed, 1–3 mg samples were sealed in pin-holed aluminium pans and heated from 25 to 250 °C at a rate of 10 °C/min. The DSC cell was purged with dry nitrogen at 80 mL/min.

A Stuart Melting Point (SMP3) apparatus (Cole-Parmer Ltd., St. Neots, UK) was used to identify melting transitions.

Infrared spectra were recorded on a Spectrum Two (PerkinElmer Inc, Waltham, MA, USA) FT-IR spectrophotometer with the Universal Attenuated Total Reflectance Accessory (UATR), over the range of 4000–400 cm^−1^ (resolution 4 cm^−1^; scans number, 4). The spectra of the precipitates were compared with that of FlAc and other excipients after baseline correction and normalisation with respect to the most intense peak of the active substance.

### 2.7. Drug Content

FlAc content was determined by adapting the HPLC method reported by El-Ragehy et al. [21]. The HPLC system consisted of an Agilent 1100 series (Agilent Technologies Inc, Santa Clara, CA, USA). The column was InertClone 5 µm ODS (3) 250 × 4.6 mm (Phenomenex Srl, Castel Maggiore, Italy). The mobile phase consisted of a phosphate buffer (pH 3.2), acetonitrile, and triethylamine 60/40/0.03 (*v*/*v*/*v*) solution, at a flow rate of 1 mL/min. The UV detector was set at 292 nm and the volume of each injection was 20 µL.

A five-point calibration curve was constructed, linearity (R^2^ > 0.999) was demonstrated in the range 10–100 µg/mL; the limit of quantification (LOQ) and limit of detection (LOD) were experimentally observed to be 0.5 and 0.1 µg/mL. Intraday and interday repeatability were tested by multiple injections of a 50 µg/mL sample and were found to be 0.07% and 0.11%, respectively. Samples were diluted with the mobile phase within the concentration interval of the standard solutions before analysis.

### 2.8. Osmolality Measurement and Microbiological Evaluation

In order to complete the characterisation of the formulations of interest (F2 and F3), osmolality was measured in the presence of 10 and 20 mg/mL FlAc. Measurements were performed in triplicate using a K-7400S freezing point osmometer (Knauer GmbH, Berlin, Germany) after dilution with ultrapure water to the calibration range (0–850 mOsm/Kg).

The 10 mg/mL FlAc solutions were also tested for microbiological stability in the presence (F3) and absence (F2) of methylparaben. Test solutions were distributed in 100 mL amber glass bottles and stored at room temperature. The stability was evaluated on days 0, 15, 30, 45 and 60 in both “after opening” (sampling made on the same bottle at each analysis time) and “before opening” (new bottle for each sampling time) conditions for the preservative-free formulation and only in “after opening” conditions for the formulation containing the preservative and used as a control. Bottles were opened in non-sterile conditions. Each analysis was performed in duplicate.

Microbiological analyses were performed according to the European Pharmacopeia monograph for non-sterile products, using the surface-spread method. European Pharmacopeia requirements indicate a total aerobic microbial count (TAMC) of less than 10^2^ CFU/mL, a total yeast and moulds count (TYMC) of less than 10^1^ CFU/mL, and the absence of *Escherichia coli* [22].

## 3. Results and Discussion

### 3.1. Solubility Study

**Figure 1:**
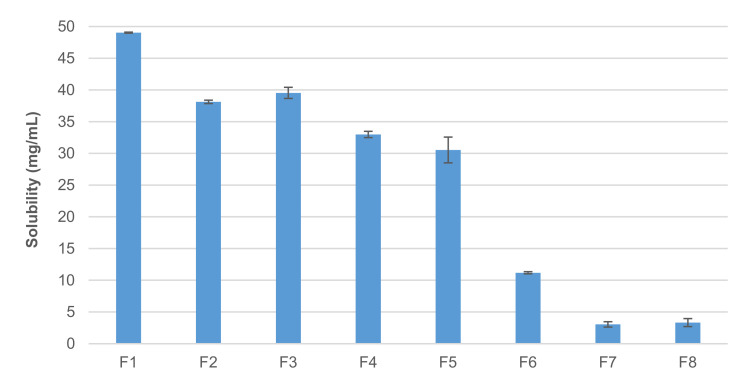
Flecainide acetate solubility in different aqueous vehicles (25 ± 1 °C). Mean ± SD (*n* = 3).

As the water solubility of the Fl free base is extremely low (0.032 mg/mL, [23]), the acetic acid salt is commonly used (about 48 mg/mL, [24]). This value was experimentally confirmed in the solubility study at 24 h. Therefore, to evaluate the possible influence of excipients (namely sweeteners, cosolvents, preservatives, and buffers), the solubility data of FlAc in the vehicles (Table 1) were determined after 24 h and reported in Figure 1. It has already been demonstrated that the presence of chloride ions can cause the precipitation of Fl due to the formation of less soluble chloride salt [25,26].

First, sucrose was considered, since it is typically used to improve the palatability of unpleasant-tasting drugs, such as FlAc [27]; moreover, it acts as an osmotic preservative against microbial contamination. Nevertheless, high consumption over long periods of time increases the risk of caries and overweight/obesity in children, as well as other possible adverse health effects [28]. It is also known that sucrose can lower the solvent power of water due to its strong hydrogen bonding capacity and hydrate shell formation; thereby, the effect of a progressively higher presence of this excipient on FlAc solubility was examined.

It was experimentally observed (Figure 1) that a 20% (*w*/*v*) sucrose content vehicle (F1) did not significantly affect the solubility of the drug substance in water, but a further increase seemed to result in a progressive reduction in solubility. With 40% sucrose (F2), in fact, saturation was reached at a lower concentration (about 38 mg/mL). Moreover, the literature reports the sugaring-out of FlAc when trying to prepare a 20 mg/mL solution in simple syrup (i.e., 85% sucrose) [15].

Based on these data, 40% sucrose was considered a good compromise between the possibility of maintaining Fl in solution and achieving a good sweetening efficacy. Previous studies evidenced the poor preservation efficacy (by challenge tests) of simple syrup when diluted with unpreserved water in ratios of 1:1 (i.e., 43% sucrose) or greater [29,30]. Therefore, methylparaben, as an antimicrobial agent, at a fixed concentration of 0.07%, was added (F3). This addition did not alter the solubility of the FlAc (Figure 1). Among parabens, methylparaben was chosen because—based on toxicological data—it appears to be the least harmful [31].

Another excipient commonly used as a co-solvent, other than mild sweetener, is glycerol. It was observed that the addition of 10% glycerol to a solution containing both sucrose and methylparaben (F4) does not improve the solubility of FlAc (Figure 1). Hence, its addition was considered unnecessary, also because its use has been related to cases of diarrhoea and electrolyte imbalance in the paediatric population [32].

According to ACD/Labs, an acidic pH should increase the solubility of FlAc [33]. Thus, the possibility of adding a buffer system at pH 4.5–5.0 to the sucrose solution was evaluated; assuming it would also be beneficial to the microbiological stability. Citrate, or, alternatively, phosphate buffers were considered, as they are the most suitable for oral administration.

Adding a citrate buffer (F5) slightly reduced FlAc solubility (Figure 1). Interestingly, a massive decrease in solubility was observed in the presence of both citrate buffer and methylparaben (F6): erratic precipitation occurred within a few minutes to hours following the addition of the excess drug substance and the solubility was three-times reduced.

The lowest FlAc solubility value was observed in the presence of phosphate buffer (F7). In this case, the solubility fell by more than 15 times (Figure 1) compared to that of pure water and did not appear to be affected by the presence of methylparaben (F8). Additional tests confirmed that the same solubility value was observed even in the sole buffer (approximately 3.0 mg/mL). It is worth noting that this huge variation in solubility could have an impact on bioavailability [34].

Lastly, in order to verify if the lack of transparency in the reported literature results [16,17,18] was only due to tablet excipients, the solubility of FlAc in two commercially available oral suspending vehicles—OraPlus^®^ and OraSweet^®^—was assessed. These both contain methylparaben, sodium phosphate and citric acid, in addition to several other excipients (as listed in the Materials paragraph). As expected, the solubility values were extremely low (2.5 ± 0.1 and 1.3 ± 0.1 mg/mL, respectively, for OraPlus^®^ and OraSweet^®^), suggesting that most of the drug is suspended. These observations are particularly relevant given that most commercially available oral suspending vehicles contain citric acid, sodium citrate, and/or sodium phosphate, as well as parabens in some cases [20]. Moreover, some of these systems are opalescent (i.e., OraPlus^®^, SyrSpend^®^), making it difficult to determine if a suspension or a solution is obtained, or if precipitation occurs later, even when the pure active substance is available.

### 3.2. Characterisation of the Precipitates

Attempts were made to isolate all the sediments obtained from the solubility tests; however, those obtained in the absence of buffers (F1–F4)—supposedly consisting of just FlAc—were completely dissolved upon the washing of the paper filter. On the other hand, the recovery and subsequent characterisation of the precipitates obtained from the buffered solutions (F5–F8) were possible.

The solid isolated by saturation in the presence of citrate ions (F5) showed a jelly-like appearance before drying and was named C1. Since the solubility of Fl is further reduced when both citrate buffer and methylparaben are present in the solution (F6), it is reasonable to assume that another different solid was obtained in these conditions. The solid was named C2 and was also obtained in the form of large, prismatic crystals during preliminary stability testing from a 10 mg/mL FlAc solution with the same vehicle.

The solid isolated from the experiments in the presence of the phosphate buffer (F7, F8) appeared as fine white powder and was named P1.

Experimental evidence supporting the hypothesis of the formation of different—yet less soluble—Fl salts in the presence of citrate or phosphate ions was provided through different analytical techniques.

First, precipitates were dissolved in the mobile phase and subjected to HPLC analysis to confirm the presence of Fl. It was calculated that the active substance accounted for 60–80% of total mass, while it should theoretically contribute for 87% when forming the acetate salt. The remaining mass fraction could be attributed in part to water—either forming hydrates or simply superficially adsorbed—and in part to one or more negatively charged counterions that may have been present in the vehicle.

For further characterisation, DSC analysis of FlAc and the three precipitates was performed in order to qualitatively assess their thermal behaviour. In addition, FT-IR spectroscopy was used to detect any anions other than acetate in the isolated solids.

**Figure 2:**
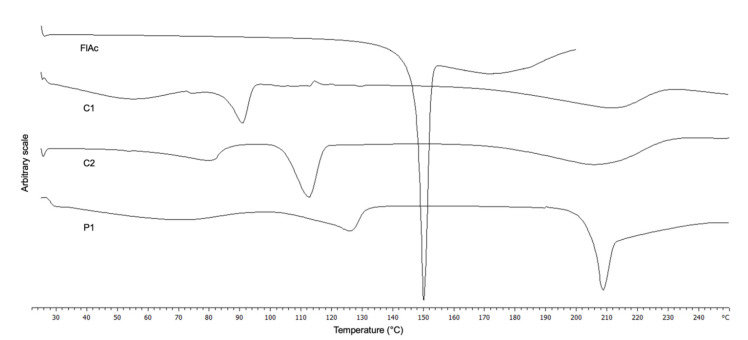
Differential scanning calorimetry curves of flecainide acetate (FlAc) and its different solid forms isolated from buffered solutions (C1 obtained from F5; C2 from F6; P1 from F7).

The DSC curves of FlAc and the three solids are shown in Figure 2. The FlAc thermogram shows a sharp endothermic signal at 150 °C which was attributed to melting; this event was immediately followed by decomposition.

Regarding C1 and C2, fusion was observed at about 91 and 112 °C, respectively, followed by decomposition over 180 °C.

**Figure 3:**
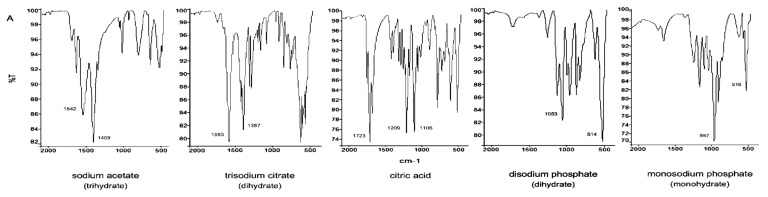

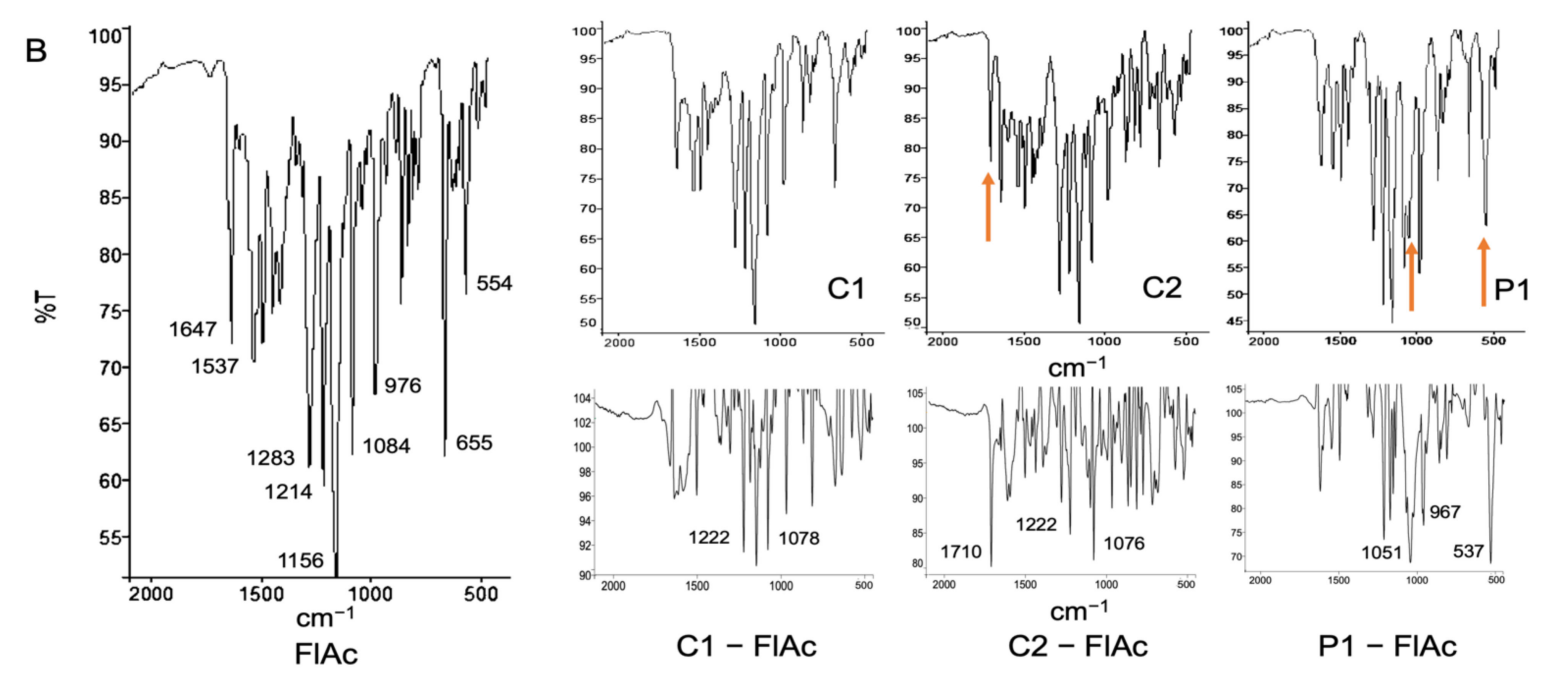
(**A**) Infrared spectra of citric acid and various sodium salts containing anions of interest; (**B**) infrared spectra and comparisons of flecainide acetate (FlAc) and its different solid forms isolated from buffered solutions (C1 obtained from F5; C2 from F6; P1 from F7).

Figure 3A illustrates the FT-IR spectra of the corresponding sodium salts of some anions of interest, in addition to citric acid. Sodium acetate and trisodium citrate are characterised by the presence of two intense absorption bands at about 1400 and 1500 cm^−1^, which are due to the symmetric and asymmetric stretching of the COO^−^ group. As opposed, undissociated carboxylic groups give a peak at about 1720 cm^−1^, resulting from C=O stretching, and a second peak at about 1200 cm^−1^, due to C-O stretching, as can be observed in the citric acid spectrum. Citric acid is also characterised by the presence of an intense peak at 1106 cm^−1^, which could be due to the C-O stretching of the tertiary alcohol group.

The FT-IR spectra of FlAc and those of the isolated solids are reported in Figure 3B. It is possible to confirm the presence of Fl in all precipitates by observing the characteristic peak pattern in the fingerprint region. The lower graphs highlight the differences among the spectra of each solid form and the pure active substance, obtained by subtracting from each spectrum that of FlAc.

It is worth noting that citric acid has three carboxylic groups; consequently, the monobasic and bibasic forms shall be present in the solution. These may show both peaks associated with carboxylic and carboxylate groups. As opposed, acetic acid possesses only one carboxylic group, therefore only the COO^−^ stretching peaks are observed in the spectra of FlAc and sodium acetate. Accordingly, an intense peak at 1710 cm^−1^ was observed in the spectra of C2, which corroborates the hypothesis of the presence of undissociated carboxylic groups contained in citric acid salts.

No other distinctive peaks were evidenced in the spectra of C1 and C2; however, it is possible to observe an increased intensity (accompanied by a slight shift) of the 1214 and 1084 cm^−1^ peaks, which is consistent with the presence of the tertiary alcohol group of the citrate, as well as of a greater number of COOH groups.

The decreased melting point and solubility and the increased decomposition temperature, together with spectroscopic data, corroborate the hypothesis of the formation of different Fl salts, other than acetate, possibly containing citrate ions.

Regarding the DSC curve of P1 (Figure 2), a melting peak is observed at 209 °C, immediately followed by endothermic decomposition. 

Considering the spectra of bibasic and monobasic sodium phosphate (Figure 3A), the first compound is characterised by the presence of a peak at 1053 cm^−1^, which corresponds to PO4^2−^ asymmetric stretching, while the second compound gives an intense peak at 957 cm^−1^, due to PO4^−^ symmetric stretching. Another characteristic band is that ranging from 500 to 600 cm^−1^, which corresponds to the out-of-plane bending modes of the phosphorous-based anions [35,36,37].

The distinctive peaks of phosphate ions can be easily identified in the P1 spectrum (Figure 3B) at 1051 and 537 cm^−1^, as indicated by the arrows, this constitutes strong evidence for the formation of a phosphate salt of Fl.

The spectroscopic data proving the presence of phosphate ions, together with the increased melting point—which is consistent with reduced solubility—confirm the formation of a phosphate salt of Fl.

### 3.3. Chemical and Physical Stability Study

The use of F1 as a vehicle was ruled out because of poor taste-masking and preservation efficacy; F7 and F8 were also dismissed, as the presence of phosphates in the solutions resulted in an excessive decrease in Fl solubility. 

**Figure 4:**
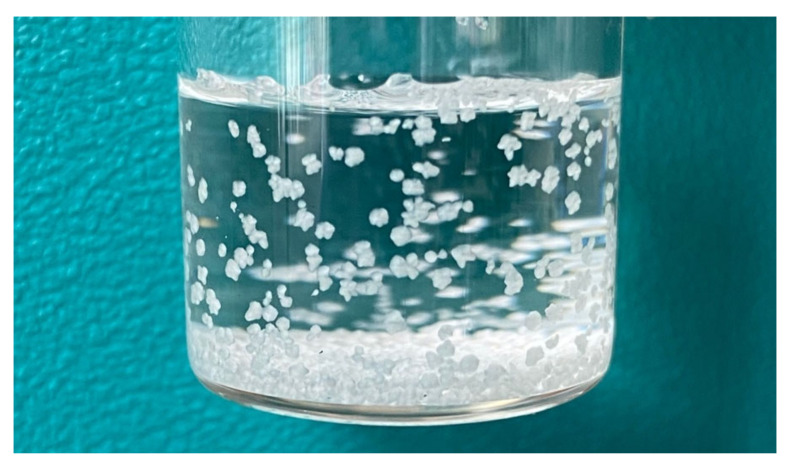
Flecainide crystals obtained from a solution containing sucrose, citrate buffer, and methylparaben (F6).

Preliminary studies on 10 mg/mL FlAc, prepared by using the vehicle solutions F2–F6, did not show any signs of physical instability within 72 h, except for F6. Immediately after compounding, the solution was clear and transparent; however, after a few days, crystals (C2) started to appear. These were attached to the container walls and could not be resuspended by manual agitation (Figure 4).

Crystal formation occurred at both refrigerator and room temperature. The supernatant was tested for FlAc content after 3 days and resulted out of specification, being about 7.5 and 6.0 mg/mL after storage at 25 and 4 °C, respectively. The evidence of a more relevant decrease in FlAc content at 4 °C—probably due to the reduced solubility—suggests that refrigeration should be avoided, if possible.

This situation is reminiscent of that described by Stuart et al. where the cause of intoxication was traced back to the crystallisation of Fl after storage in a refrigerator (unfortunately, the composition of the suspending vehicle was not reported) [19]. In fact, the administration of an aliquot of a solution containing floaters of concentrated Fl may result in over-dosing rather than under-dosing.

It is worth noting that crystal formation was not observed in 10 mg/mL FlAc solutions containing only citrate buffer (F5) or preservative (F3, F4).

Based on all the above-reported observations, F3 was chosen as the most appropriate vehicle for the oral administration of FlAc. The strengths considered for the stability assessment under different storage conditions were 10 and 20 mg/mL.

FlAc was chemically stable at both concentrations for 8 weeks under all storage conditions (Table 2). The pH of the solutions was close to neutrality (ranging from 6.4 to 6.7) and remained constant for the duration of the study. Moreover, no precipitate formation or any other signs of physical instability were observed, confirming that the sole presence of methylparaben does not compromise the solubilisation of the active substance.

### 3.4. Osmolality and Microbiological Evaluation

Considering the 10 mg/mL strength, the osmolality was 1.282 ± 10 and 1.307 ± 3 mOsm/Kg for F2 and F3, respectively. In the case of the 20 mg/mL solutions, osmolality values were found to be slightly higher, reaching 1.325 ± 5 and 1.372 ± 3 mOsm/Kg, respectively. Although these values exceed the maximum osmolality limit recommended for paediatric formulae (450 mOsm/kg) [38], they are relatively low compared to those observed for other oral medications commonly administered to neonates [39].

Despite parabens being the most commonly used preservatives, there are several health concerns related to their use. In fact, a number of studies demonstrated the link between exposure to parabens and endocrine-disrupting effects, with particular consequences on the concentrations of sex hormones and thyroid hormones [40]. For this reason, the European Medicines Agency (EMA) recommends avoiding the use of preservatives wherever possible, especially in the case of paediatric formulations; when necessary, the concentration used should be the lowest practicable [41]. Accordingly, this study also aimed to assess the actual need for a preservative to be added to the proposed formulation by recreating in-use conditions.

According to the microbiological stability assessment, comparing solutions of FlAc 10 mg/mL with (F3) and without the preservative (F2), both formulations, in all the evaluated conditions, complied with the European Pharmacopoeia specifications on the microbial examination of non-sterile products over 60 days.

## 4. Conclusions

As the paediatric population from premature neonate to adolescent is very heterogeneous, it cannot be approached as a uniform group. This brings practical issues in the design of dosage forms, regarding dose flexibility, delivery of the correct dose, and patient/caregiver acceptability. Further considerations involve formulation properties such as dosage strength, solubility, taste, and stability; therefore, specific attention is paid to the choice of excipients. The EMA has offered some guidance on the selection of dosage forms and excipients in relation to acceptability by paediatric patients, besides a hierarchised list of information sources to consult in order to assess the safety profile of each component. However, the approach “less is more” should be followed whenever possible. Indeed, the excipients commonly used in the formulation of oral liquid vehicles can lead to drug precipitation—not noticeable if the vehicle is opaque—which may negatively affect the safety and efficacy of the treatment.

In the case of FlAc, a conversion to less soluble salts was demonstrated to occur in the presence of other salts such as citrates and phosphates. Their combination with methylparaben may even lead to the development of large non-resuspendable crystals, which can be the cause of dosing errors. The main concern is the erratic formation over time; because of this, the unaware compounding pharmacist may dispense the preparation without noticing the problem. This set of data demonstrated that 10 and 20 mg/mL FlAc is chemically, physically, and microbiologically stable for over 8 weeks at room temperature when compounded by using a (40%) sucrose solution.

Besides providing robust documentation on the drug’s stability and compatibility, this study confirms that careful experimental work supporting the development of extemporaneous preparations is essential to avoid unforeseen events and could be of help for a new compendial monograph concerning this pharmacy preparation.

## Figures and Tables

**Table 1:** Composition (g) of aqueous vehicles intended for the oral delivery of flecainide acetate.

	F1	F2	F3	F4	F5	F6	F7	F8
Sucrose	20	40	40	40	40	40	40	40
Methylparaben	-	-	0.07	0.07	-	0.07	-	0.07
Glycerol	-	-	-	10	-	-	-	-
Citric acid	-	-	-	-	0.1	0.1	-	-
Sodium citrate	-	-	-	-	0.08	0.08	-	-
Monosodium phosphate	-	-	-	-	-	-	0.21	0.21
Sodium hydroxide (1M) *	-	-	-	-	-	-	q.s.	q.s.
Water **	87.5	74.5	74.5	66.5	74.5	74.5	74.5	74.5

* q.s. to pH 4.5–5.0; ** the amount of water is added to reach a total volume of 100 mL.

**Table 2:** Stability data of flecainide acetate oral solutions at different temperatures using F3 as a vehicle. Mean ± SD (*n* = 3).

Storage Temperature	Actual Initial Concentration (mg/mL)	% Labeled Concentration Remaining			
		14 Days	28 Days	42 Days	56 Days
10 mg/mL flecainide acetate oral solution					
4 °C	10.3 ± 0.0	102 ± 2	102 ± 1	101 ± 3	100 ± 1
25 °C		104 ± 1	103 ± 2	100 ± 1	100 ± 2
40 °C		103 ± 2	104 ± 1	102 ± 4	103 ± 4
20 mg/mL flecainide acetate oral solution					
4 °C	20.1 ± 0.0	102 ± 2	102 ± 3	98 ± 1	101 ± 1
25 °C		102 ± 1	101 ± 2	99 ± 1	100 ± 1
40 °C		102 ± 1	102 ± 2	98 ± 1	102 ± 0

## Data Availability

The data presented in this study are available on request from the corresponding author.

## References

[1] Rodieux F., Vutskits L., Posfay-Barbe K.M., Habre W., Piguet V., Desmeules J.A., Samer C.F. (2018). When the Safe Alternative Is Not That Safe: Tramadol Prescribing in Children. Front. Pharmacol..

[2] Gore R., Chugh P.K., Tripathi C.D., Lhamo Y., Gautam S. (2017). Pediatric Off-Label and Unlicensed Drug Use and Its Implications. Curr. Clin. Pharmacol..

[3] Casiraghi A., Musazzi U.M., Franceschini I., Berti I., Paragò V., Cardosi L., Minghetti P. (2014). Is Propranolol Compounding from Tablet Safe for Pediatric Use? Results from an Experimental Test. Minerva Pediatr..

[4] Council of Europe European Resolution CM/Res(2016)1 on Quality and Safety Assurance Requirements for Medicinal Products Prepared in Pharmacies for the Special Needs of Patients. https://search.coe.int/cm/Pages/result_details.aspx?ObjectID=090000168065c132.

[5] (2008). Norme Di Buona Preparazione Dei Medicinali in Farmacia. Farmacopea Ufficiale Della Repubblica Italiana.

[6] Minghetti P., Pantano D., Gennari C.G.M., Casiraghi A. (2014). Regulatory Framework of Pharmaceutical Compounding and Actual Developments of Legislation in Europe. Health Policy.

[7] Zanon D., Selmin F., Centin G., Maximova N., Casiraghi A., Minghetti P. (2021). Stability of High Concentrated Triple Intrathecal Therapy for Pediatrics and Mitigation Strategies. Eur. J. Pharm. Sci..

[8] Casiraghi A., Musazzi U.M., Rocco P., Franzè S., Minghetti P. (2016). Topical Treatment of Infantile Haemangiomas: A Comparative Study on the Selection of a Semi-Solid Vehicle. Skin Pharmacol. Physiol..

[9] Casiraghi A., Gennari C.G., Musazzi U.M., Ortenzi M.A., Bordignon S., Minghetti P. (2020). Mucoadhesive Budesonide Formulation for the Treatment of Eosinophilic Esophagitis. Pharmaceutics.

[10] Ogawa R., Stachnik J.M., Echizen H. (2014). Clinical Pharmacokinetics of Drugs in Patients with Heart Failure: An Update (Part 2, Drugs Administered Orally). Clin. Pharmacokinet..

[11] Perry J.C., Garson A. (1992). Flecainide Acetate for Treatment of Tachyarrhythmias in Children: Review of World Literature on Efficacy, Safety, and Dosing. Am. Heart J..

[12] Cunningham T., Uzun O., Morris R., Franciosi S., Wong A., Jeremiasen I., Sherwin E., Sanatani S. (2017). The Safety and Effectiveness of Flecainide in Children in the Current Era. Pediatr. Cardiol..

[13] (2016). British National Formulary for Children 2016–2017.

[14] Haywood A., Glass B.D. (2013). Liquid Dosage Forms Extemporaneously Prepared from Commercially Available Products—Considering New Evidence on Stability. J. Pharm. Pharm. Sci..

[15] Santoveña A., Charola I., Suárez-González J., Teigell-Pérez N., García-van Nood S., Soriano M., Fariña J.B. (2018). Development of a Novel Physico-Chemically and Microbiologically Stable Oral Solution of Flecainide for Pediatrics. Pharm. Dev. Technol..

[16] Wiest D.B., Garner S.S., Pagacz L.R., Zeigler V. (1992). Stability of Flecainide Acetate in an Extemporaneously Compounded Oral Suspension. Am. J. Hosp. Pharm..

[17] Allen L.V.J., Erickson M.A. (1996). Stability of Baclofen, Captopril, Diltiazem Hydrochloride, Dipyridamole, and Flecainide Acetate in Extemporaneously Compounded Oral Liquids. AJHP Off. J. Am. Soc. Health Pharm..

[18] Uriel M., Gómez-Rincón C., Marro D. (2018). Stability of Regularly Prescribed Oral Liquids Formulated with SyrSpend(^®^) SF. Pharmazie.

[19] Stuart A.G., Wren C., Bain H.H. (1989). Is There a Genetic Factor in Flecainide Toxicity?. Br. Med. J..

[20] Helin-Tanninen M., Autio K., Keski-Rahkonen P., Naaranlahti T., Järvinen K. (2012). Comparison of Six Different Suspension Vehicles in Compounding of Oral Extemporaneous Nifedipine Suspension for Paediatric Patients. Eur. J. Hosp. Pharm. Sci. Pract..

[21] El-Ragehy N.A., Hassan N.Y., Tantawy M.A., Abdelkawy M. (2016). Stability-Indicating Chromatographic Methods for Determination of Flecainide Acetate in the Presence of Its Degradation Products; Isolation and Identification of Two of Its Impurities. Biomed. Chromatogr..

[22] (2017). European Pharmacopoeia.

[23] Flecainide. https://hmdb.ca/metabolites/HMDB0015326.

[24] Flecainide. https://go.drugbank.com/drugs/DB01195.

[25] Woods D.J. Flecainide Acetate. http://www.fshealth.gov.za/subsites/DWoods/Mixtures/flecainide.html.

[26] SA Health South Australian Neonatal Medication Guidelines—Flecainide. https://www.sahealth.sa.gov.au/wps/wcm/connect/dcc5f7d4-7c6e-46b5-a6a7-6cd7f249e7da/Flecainide_Neo_v1_0.pdf?MOD=AJPERES&CACHEID=ROOTWORKSPACE-dcc5f7d4-7c6e-46b5-a6a7-6cd7f249e7da-nGtHHWO.

[27] Tessarolo Silva F., Pedreira G.C., Medeiros S.A., Bortolotto A.L., Araujo Silva B., Hurrey M., Madhavapeddi P., Schuler C., Belardinelli L., Verrier R.L. (2020). Multimodal Mechanisms and Enhanced Efficiency of Atrial Fibrillation Cardioversion by Pulmonary Delivery of a Novel Flecainide Formulation. J. Cardiovasc. Electrophysiol..

[28] Fidler Mis N., Braegger C., Bronsky J., Campoy C., Domellöf M., Embleton N.D., Hojsak I., Hulst J., Indrio F., Lapillonne A. (2017). Sugar in Infants, Children and Adolescents: A Position Paper of the European Society for Paediatric Gastroenterology, Hepatology and Nutrition Committee on Nutrition. J. Pediatr. Gastroenterol. Nutr..

[29] Santoveña-Estévez A., Suárez-González J., Vera M., González-Martín C., Soriano M., Fariña J.B. (2018). Effectiveness of Antimicrobial Preservation of Extemporaneous Diluted Simple Syrup Vehicles for Pediatrics. J. Pediatr. Pharmacol. Ther..

[30] Ghulam A., Keen K., Tuleu C., Wong I.C.-K., Long P.F. (2007). Poor Preservation Efficacy versus Quality and Safety of Pediatric Extemporaneous Liquids. Ann. Pharmacother..

[31] European Medicines Agency (2015). Reflection Paper on the Use of Methyl- and Propylparaben as Excipients in Human Medicinal Products for Oral Use.

[32] Yellepeddi V., Vangara K. (2015). Excipients in Pediatric Formulations: Biopharmaceutical and Toxicological Considerations. Excipient Applications in Formulation Design and Drug Delivery.

[33] Flecainide—Substance Detail, Predicted Properties (Calculated using Advanced Chemistry Development Software V11.02, © 1994–2021 ACD/Labs). https://scifinder-n.cas.org/searchDetail/substance/6133810cb914160d7af8bb26/substanceDetails.

[34] Amidon G.L., Lennernäs H., Shah V.P., Crison J.R. (1995). A Theoretical Basis for a Biopharmaceutic Drug Classification: The Correlation of in Vitro Drug Product Dissolution and in Vivo Bioavailability. Pharm. Res..

[35] Trivedi M., Branton A., Trivedi D., Nayak G., Bairwa K., Jana S. (2015). Spectroscopic Characterization of Disodium Hydrogen Orthophosphate and Sodium Nitrate after Biofield Treatment. J. Chromatogr. Sep. Tech..

[36] Shanmugam S.D., Kulandaivelu R., Tsn S.N., Lee M.H. (2014). A Facile Electrochemical Approach for the Deposition of Iron-Manganese Phosphate Composite Coatings on Aluminium. RSC Adv..

[37] Carella F., Degli Esposti L., Barreca D., Rizzi G.A., Martra G., Ivanchenko P., Escolano Casado G., Gomez Morales J., Delgado Lòpez J.M., Tampieri A. (2019). Role of Citrate in the Formation of Enamel-like Calcium Phosphate Oriented Nanorod Arrays. CrystEngComm.

[38] Barness L.A., Mauer A.M., Holliday M.A., Anderson A.S., Dallman P.R., Forbes G.B., Goldbloom R.B., Haworth J.C., Jesse M.J., Scriver C.R. (1976). Commentary on Breast-Feeding and Infant Formulas, Including Proposed Standards for Formulas. Pediatrics.

[39] Shah D.D., Kuzmov A., Clausen D., Siu A., Robinson C.A., Kimler K., Meyers R., Shah P. (2021). Osmolality of Commonly Used Oral Medications in the Neonatal Intensive Care Unit. J. Pediatr. Pharmacol. Ther. JPPT Off. J. PPAG.

[40] Nowak K., Ratajczak-Wrona W., Górska M., Jabłońska E. (2018). Parabens and Their Effects on the Endocrine System. Mol. Cell. Endocrinol..

[41] European Medicines Agency (2007). Guideline on Excipients in the Dossier for Application for Marketing Authorisation of a Medicinal Product.

